# Spontaneous Isolated Infection of the Subacromial Bursa

**DOI:** 10.1155/2013/564690

**Published:** 2013-07-29

**Authors:** Hayat Khan, Karam Al-Tawil

**Affiliations:** Orthopaedics, Darent Valley Hospital, Darenth Wood Road, Dartford, Kent DA2 8DA, UK

## Abstract

Isolated infection of the subacromial bursa is a rare entity. We present the case of a previously fit man who was found to have staphylococcal infection of the sub-acromial bursa, without an obvious precipitant. Preoperative MRI scanning determined the specific locus of infection, and the patient was successfully treated with arthroscopic washout of the sub-acromial bursa followed by empirical antibiotic therapy.

## 1. Introduction

The subacromial bursa is the synovial membrane located just below the acromion. The capsule extends above the humeral head to form a bursa between the humeral head and the overlying acromial process; this is often the site of pathology in the form of impingement and is often injected or arthroscopically debrided, though rarely infected.

## 2. Case Presentation

**Figure 1:**
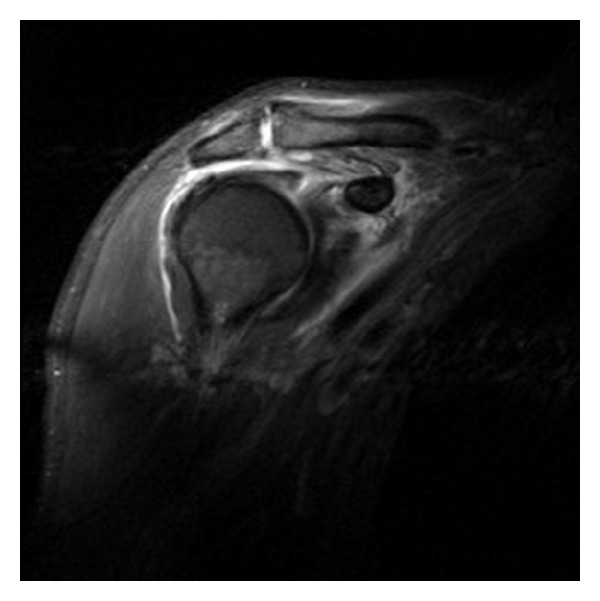
Coronal T2-weighted MRI scan showing collection in subacromial bursa.

**Figure 2:**
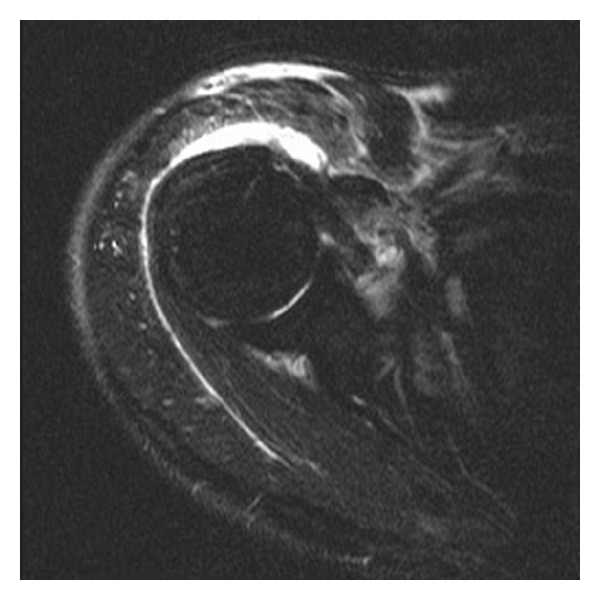
Axial T2-weighted MRI scan showing collection in anterior aspect of subacromial bursa.

A sixty-two-year-old, right-hand-dominant man presented with a 5-day history of progressive pain and swelling of his right shoulder and a 48-hour history of fever and rigors. There was no history of trauma. The patient was previously fit and well, with no specific prior shoulder pathology. On examination, the patient was pyrexial with a temperature of 38 degrees. General examination was unremarkable. On inspection, the right shoulder was swollen, with erythema in the anterior and superior shoulder regions. This area was warm to touch, with acute tenderness in the subacromial region. The patient was unable to actively move his shoulder, and minimal passive movement elicited severe pain. The acromioclavicular joint was not directly tender. The white cell count (WCC) was 13.6 × 10^9^/L, and the C Reactive Protein (CRP) was 400 mg/L. T2-weighted MRI of the shoulder revealed a high signal collection confined to the subacromial bursa, with reactive high signal in the subdeltoid bursa and acromio-clavicular joint (Figures 1 and 2). At arthroscopy pus was aspirated from the subacromial space, which was then debrided and irrigated thoroughly with 6 litres of normal saline (without antibiotic or antiseptic derivative). The glenohumeral joint was not explored; this was based upon its normal appearances on the MRI images and for fear of contaminating the joint.

Postoperatively the patient had immediate improvement in his symptoms and range of movement in his shoulder. Blood results three days after admission revealed a white cell count of 11.4–13.6 × 10^9^/L and a CRP of 179 mg/L. He was treated with an empirical course of intravenous benzylpenicillin, flucloxacillin, and gentamicin, which was later changed to oral flucloxacillin upon discharge for a total of six weeks. The intraoperative aspirate was positive for *Staphylococcus aureus*, which was sensitive to the penicillin-based antibiotics.

At the six-week follow-up appointment, the patient was well with no problems relating to the right shoulder. On examination he was nontender and had a full range of active movement of his shoulder. His white cell count and CRP had returned to normal levels.

## 3. Discussion

Septic bursitis is commonly seen in superficial bursae, such as the olecranon or the bursae around the knee. These sites are easily inoculated transcutaneously through minor trauma [1, 2]. Infection of the subacromial bursa is uncommon, with only a few such cases in the literature. Most of the reported cases involved immunocompromised patients or a known route of infection. In the case we describe, the patient was fit and well; there was no history of trauma, and he was not immunocompromised. A multitude of organisms have been found to be the cause of infection of the superficial bursae. The subacromial bursa, by contrast, is deep and therefore less likely to be infected by the transcutaneous route [1–3]. 

Routes of infection into the subacromial bursa are associated with three factors: (i) in immunocompromised individuals (e.g., hepatic or renal failure and neoplastic disease) [2]; (ii) injection into the subacromial bursa (typically for rotator cuff tendonitis and impingement syndrome) [4]; and (iii) by haematogenous spread (e.g., intravenous drug abuse, tuberculosis) [1, 5].

Ward and Eckardt (1993) describe a series of four chronically ill patients with existing shoulder polyarthrosis with subacromial/subdeltoid bursa abscesses; here *staphylococcus aureus* was the implicated organism [3]. Co and Baer (1990) reported the case of an intravenous drug user who developed a staphylococcal aureus infection of the subacromial/subdeltoid bursa. This was diagnosed via bursography, instillation of radiocontrast dye into the infected cavity [1]. Khazzam et al., 2005, describe a single patient developing candida infection of the subacromial bursa, following multiple corticosteroid injections into an injured shoulder. At arthroscopy there was substantial proliferation and inflammation of the subacromial bursa. In addition there was also inflammation of the glenohumeral joint. This patient was successfully treated with complete resection of the subacromial bursa [2].

Chartash et al. (1992), describe four cases of septic subdeltoid arthritis, all involving immunocompromise, trauma, or prior shoulder treatment. MRI was used in these cases, but after aspiration or formal incision and drainage, to determine the extent of bursal involvement and the need for further treatment [6].

With all of these previously reported cases, there were obvious precipitants to subacromial bursal infection. In our case there was no history of systemic illness or immunocompromise and no history of trauma or injection into the shoulder region. A potential criticism of our management in this case is to ask why the glenohumeral joint was not explored and/or washed out. The answer to this lies in the specific focus of the patient's pain and tenderness in relation to the subacromial bursa, as well as the specific nature of the high MRI signal in the corresponding region. We felt that the risk of entering the glenohumeral joint outweighed the risk of missing pathology. There may have been a reactive inflammation within the glenohumeral joint, but this was still sterile, and this clearly settled as the patient's subacromial infection was treated. 

We have highlighted an interesting case in a fit patient, with *isolated* infection of the subacromial bursa. Furthermore, in comparison to previously reported cases on subacromial bursal infection, we have used MRI as our main radiological investigation in the acute phase to help guide focused treatment.
